# Precision cardio-oncology: understanding the cardiotoxicity of cancer therapy

**DOI:** 10.1038/s41698-017-0034-x

**Published:** 2017-09-12

**Authors:** Xinqiang Han, Yun Zhou, Wendi Liu

**Affiliations:** 1 Reid Health, Indiana University School of Medicine, Richmond, IN 47374 USA; 2 Professor of Medicine and Director, Cancer Center of Henan Provincial People’s Hospital, Zhengzhou University, Zhengzhou, Henan China; 3 Professor of Biochemistry and Director, Zhengzhou Central Laboratory of Antibody Research, Henan University of Traditional Chinese Medicine, Zhengzhou, Henan China

## Abstract

Current oncologic treatments have brought a strong reduction in mortality in cancer patients. However, the cancer therapy-related cardiovascular complications, in particular chemo-therapy and radiation therapy-induced cardiotoxicities are a major cause of morbidity and mortality in people living with or surviving cancer. The simple fact is that all antineoplastic agents and radiation therapy target tumor cells but also result in collateral damage to other tissues including the cardiovascular system. The commonly used anthracycline chemotherapy agents can induce cardiomyopathy and congestive heart failure. Targeted therapies with human epidermal growth factor antibodies, tyrosine kinase inhibitors or vascular endothelial growth factor antibodies, and the antimetabolites also have shown to induce cardiomyopathy and myocardial ischemia. Cardiac arrhythmias and hypertension have been well described with the use of tyrosine kinase inhibitors and antimicrotubule agents. Pericarditis can happen with the use of cyclophosphamide or cytarabine. Mediastinal radiation can cause constrictive pericarditis, myocardial fibrosis, valvular lesions, and coronary artery disease. Despite significant progresses in the understanding of the molecular and pathophysiologic mechanisms behind the cardiovascular toxicity of cancer therapy, there is still lack of evidence-based approach for the monitoring and management of patients. This review will focus mainly on the recent advances in the molecular mechanisms of cardiotoxicity related to common cancer therapies while introducing the concept of cardio-oncology service. Applying the general principles of multi-disciplinary approaches toward the diagnosis, prevention, monitoring, and treatment of cancer therapy-induced cardiomyopathy and heart failure will also be discussed.

## Introduction

Heart disease and cancer are the top two causes of mortality globally, accounting for 46.1% of deaths worldwide.^1, 2^ Cardiovascular complications of cancer therapy significantly contribute to the global burden of cardiovascular disease (CVD). Congestive heart failure (CHF) in particular is a relatively common and life-threatening complication. While contemporary cancer treatment truly represents a medical success story because 5-year survival rates for all malignancies have increased from 50% in the 1975–1997 period to 68% in the 1998–2005 period,^3^ this success has produced a large cohort of cancer survivors with increased risk of chronic multi-systemic diseases.^4^ In 2014 there were ~14.5 million American cancer survivors^5^ and the number is anticipated to reach 18 million by 2020.^6^ In Europe ~3 million patients are diagnosed with cancer each year, which means there is a large group at risk of treatment-related complications.^7^ Improved survival is often accompanied by treatment-related complications, including adverse effects of cancer therapies on the heart. Cancer therapies including cytotoxic chemotherapies, molecularly targeted therapies, and mediastinal irradiation have been linked to myocyte damage, left ventricular systolic and diastolic dysfunctions, CHF, thrombogenesis, pericardial disease, hypertension, myocardial ischemia, cardiac arrhythmias, and vasospasm.^8, 9^ In particular, CHF as a result of cancer therapy has been linked to a 3.5-fold increased mortality risk compared with idiopathic cardiomyopathy.^10^ In the long term, the risk of death from CVD may exceed the risk of recurrence for many forms of cancer.^11, 12^

**Fig. 1:**
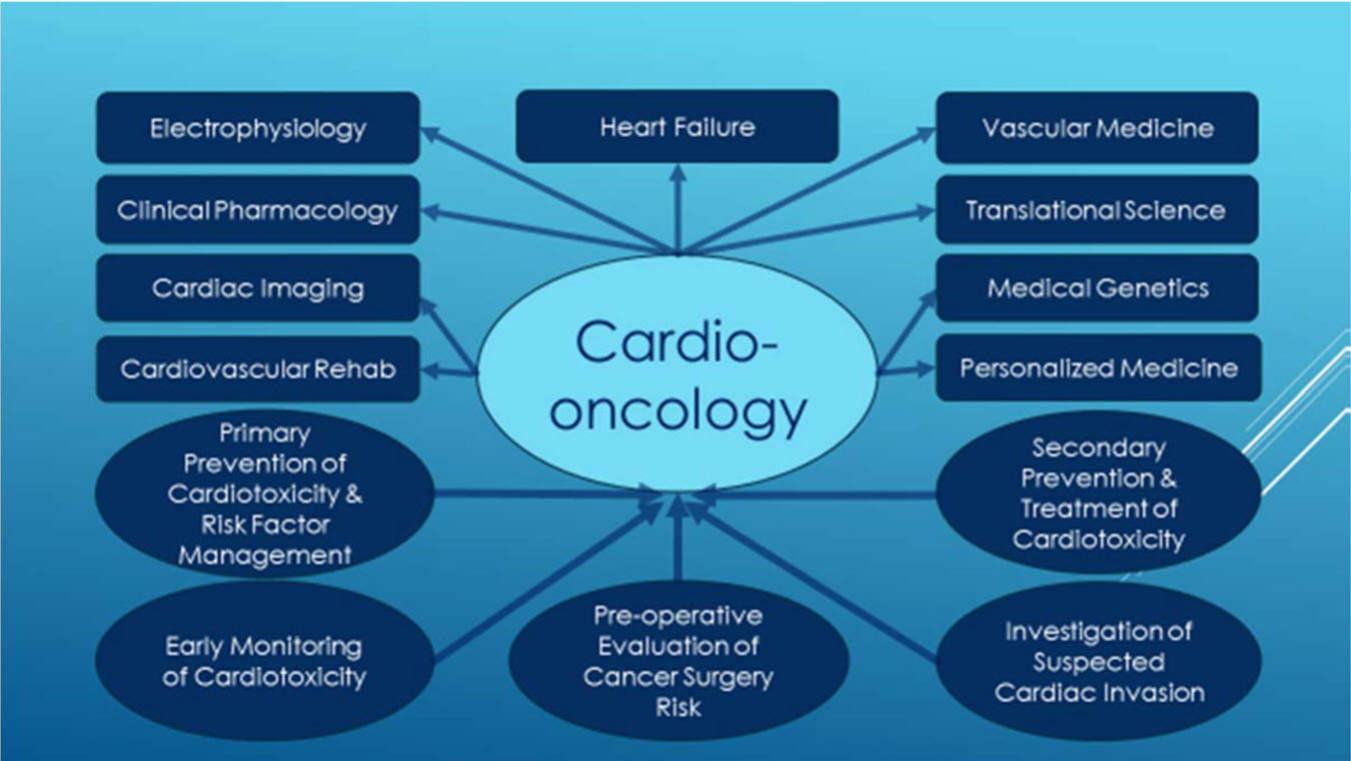
Cardio-oncology service with major interactive specialties and common referrals

For most cardiologists the CVD of cancer survivors are managed just like the patients with chronic comorbidity such as diabetes or hypertension rather than a terminal illness, except such managements can be considerably more challenging. Not infrequently, when a cardiac patient develops a malignancy the cardiologist loses interest for pursuing further diagnosis that may lead to appropriate intensive treatment and/or intervention possibilities. Conversely, failure to predict the long-term consequences of cancer treatment–associated cardiovascular complications leads to under-diagnosis or over-diagnosis of CVD, sometimes resulting in ineffective prevention of the adverse events and sometimes to inappropriate interruption of a potentially lifesaving treatment. As a consequence the management of those patients may be inadequate, and most importantly, the patients feel left alone and unprotected. Adding to the complexity is the ever-expanding number of cancer therapies targeting novel kinases, as well as other specific cellular and metabolic pathways that are being developed and tested in oncology clinical trials. Some of these drugs may impact the cardiovascular system in detrimental means while others perhaps in beneficial ways. Despite development of the new interdisciplinary area of cardio-oncology within the past two decades,^13^ patients demand and deserve better quality of care from cardiologists and oncologists. While there is no perfect definition, the term cardio-oncology or onco-cardiology we use in this paper describes the integrative and translational medicine between cardiologists and oncologists focusing on the diagnosis, prevention, and management of cardiovascular complications associated with the development and treatment of malignancy. A schematic drawing of the current cardio-oncology service with its interactive subspecialties, as well as major referrals is illustrated in Fig. 1 which will be referred to and discussed throughout the review. In the era of personalized or precision medicine with exploding information from translational investigations of molecular and genetic targets, close interactions between the two specialties are mandatory for the optimization of anti-cancer therapies, cardiovascular complication prevention, and drug discovery. The following discussion will focus mainly on the molecular mechanisms of common cancer therapy related cardiotoxicity and the principles of multi-disciplinary approaches to the diagnosis, prevention, monitoring, and treatment of the cardiovascular complications related to cancer therapy. A detailed review of the precision oncology aspects of the cancer therapy agents is beyond the scope of this manuscript, and wherever appropriate and relevant, updated references will be cited.

## Cardiovascular complications of cancer therapy: molecular targeting

The most recent ESC guideline broadly divides the cardiovascular complications of cancer therapy into nine major categories^14^ pertaining to either the cardiac or the vascular system. The cardiac complications encompass myocardial dysfunction and CHF, coronary artery disease, valvular heart disease, arrhythmias, and pericardial diseases. The vascular complications would include arterial hypertension, thromboembolic event, peripheral vascular disease and stroke, and pulmonary hypertension. The cardiac toxicity of antineoplastic agents can be addressed from four aspects: direct cytotoxic effects of chemotherapy and associated myocardial dysfunction, cardiac ischemia, cardiac arrhythmias, and pericarditis. Radiation therapy can also lead to coronary artery disease and fibrotic changes to the valves, pericardium, and myocardium. In the following we will focus our discussions on the cardiotoxicity relating to different classes of cancer therapies and the underlying cellular, as well as molecular mechanisms which are briefly summarized in Table 1. Readers are also referred to a number of excellent reviews summarizing the cardiovascular toxic effects of the targeted immunotherapies.^15, 16^

**Table 1:** Common anticancer therapies and their molecular mechanisms of cardiotoxicity

Anticancer therapies	Molecular mechanisms of cardiotoxicity
Anthacyclines	Activate Necleus TopIIβ (inhibited by Dexrazoxane)
	Generate ROS
	Activate TopImt
	Fe^2+^ overload (chealated by Dexrazoxane)
	Damage transcription
	Energy depletion
	Prevent DNA repair
Alkylating agents	Cause endothelial dysfunction
	Cause thrombosis
	Direct DNA damage
HER2/ERB2 Ab	Inhibit Pro-Survival NRG-1/ErbB Pathway
	Generate ROS
TKIs/VEGFR Ab	Inhibit angiogenesis
	Cause endothelial dysfunction
	Cause energy depletion
Antimetabolites	Inhibit angiogenesis
	Cause endothelial dysfunction
	Cause energy depletion
	Generate ROS
Antimicrotubules	Inhibit microtubule formation
	Activate NCS-1 causing Ca^2+^ overload
Radiation therapy	Inhibit angiogenesis
	Cause endothelial dysfunction
	Cause energy depletion
	Generate ROS

*TKIs* tyrosine kinase inhibitors, *VEGFR* vascular endothelial growth factor receptor, *NRG-1* neuregulin-1, *HER2/ErbB2* human epidermal growth factor receptor 2, *Ab* antibody, *TopImt* mitochondrial topoisomerase I, *TopIIβ* topoisomerase IIβ, *ROS* reactive oxygen species, *NCS-1* neuronal calcium sensor 1

### Anthracycline cardiotoxicity

Elucidation of the cellular and molecular mechanisms of anthracycline cardiotoxicity takes time and multi-disciplinary efforts. Daunorubicin was the first anthracycline with cardiotoxicity being reported half a century ago.^17^ In the original study, of the 19 children of solid tumors or acute leukemia who received daunorubicin at a cumulative dose of 25 mg/kg and higher 14 died after 1 week up to 9 months. “Seven, prior to death, developed cardiopulmonary symptoms characterized by tachycardia, with or without arrhythmia, gallop rhythm and in some cases CHF, tachypnea and in some cases dyspnea”.^17^ The discovery and application of other anthracycline chemotherapies and the demonstration of dose-dependent probability of CHF in the 1970s^18, 19^ were perhaps the first event to foster partnership between oncologists and cardiologists. Since then, efforts to reduce anthracycline-induced cardiotoxic effects through dose limitation, chemical protection, change in formulation, or change in delivery schedule have been consistently made.^20^ Two types of cardiotoxicity were described: acute reaction which usually occurs during or shortly after chemotherapy and chronic response which develops months, years, or decades after completion of therapy. Acute cardiotoxicity mainly presented as electrocardiographic changes and/or cardiac arrhythmias.^17, 21–24^ The incidence of CHF as a result of chronic toxicity of anthracycline treatment varied from 3–30%, largely owing to differences in the patient populations studied, clinical criteria including systolic and diastolic or both defined, and accumulative doses used.^17, 21–24^ Myocardial injury can occur unpredictably with doses as low as 200 mg/m^2^, and the incidence increases steeply as doses exceed 550 mg/m^2^. The delayed manifestation of chronic cardiotoxicity can be a significant challenge as many patients might not receive regular preventive surveillance or timely management at the early stage of disease development which may be reversible with effective interventions. Currently there is still a lack of guideline or standardized risk stratification recommendation despite a growing number of cancer survivors are at increased lifetime risk of anthracycline-induced cardiotoxicity.

**Fig. 2:**
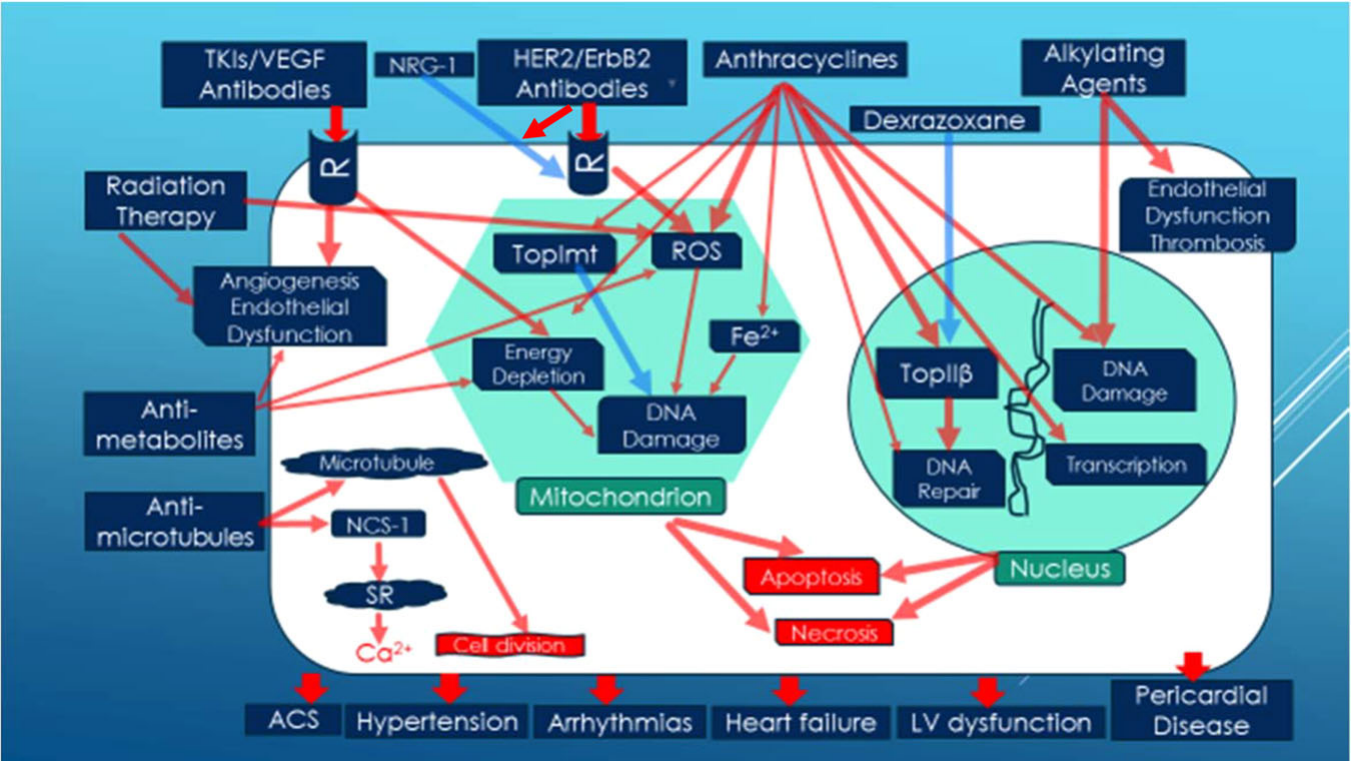
Mechanisms of cardiovascular injuries from commonly used cancer therapies. Common cellular targets and pathophysiological pathways are schematically illustrated. *Red arrows* denote detrimental effects; *Blue arrows* imply protective effects. The eventual death of cardiomyocytes and endothelial dysfunction lead to various cardiovascular complications. Refer to text for detail. *Abbreviations*: *TKIs* tyrosine kinase inhibitors, *VEGF* vascular endothelial growth factor, *NRG-1* neuregulin-1, *HER2/ErbB2* human epidermal growth factor receptor 2, *R* receptor, *TopImt* mitochondrial topoisomerase I, *TopIIβ* topoisomerase IIβ, *ROS* reactive oxygen species, *NCS-1* neuronal calcium sensor 1, *SR* sarcoplasmic reticulum, *ACS* acute coronary syndrome

Several mechanisms have been proposed to explain the anthracycline (using doxorubicin, Dox, as an example) cardiotoxicity but one of the commonly accepted explanations is the oxidative stress which generates reactive oxygen species (ROS) and oxygen free radicals (OFR) during oxidative respiratory chain reaction in the mitochondria. Metabolism of Dox can generate excessive ROS such as superoxide, hydrogen peroxide, the reactive nitrogen species (RNS), as well as other OFR that are far beyond the clearance capacity of the antioxidant-producing enzymes (such as peroxidase, catalase, and superoxide dismutase) and the metabolism by NADPH dehydrogenase, cytochrome P-450 reductase, and xanthine oxidase. The resulting damages to DNA, RNA, proteins, and membrane lipids from ROS, RNS and OFR lead to cardiomyocyte death. Apoptosis, necrosis, and autophagy all can be involved during generation of ROS and lipid peroxidation but the role of autophagy in Dox-mediated cell death is still controversial.^24, 25^ Both ROS and RNS can affect ion channel proteins including several K^+^ currents causing action potential propagation abnormality and cardiac arrhythmias.^26^ OFR also inhibits calcium sensor proteins in the excitation-contraction coupling causing myocardial stunning.^27^ Heart as a pumping organ would require large amount of energy generation and turnover to support its physiological function. As such the mitochondria which are rich in cardiac myocytes has been identified as the major subcellular target in doxorubicin-induced cardiotoxicity (see Fig. 2). Iron has been shown to play an important role in this process.^28, 29^ Dox is known to chelate free iron to form iron–Dox complexes which react with oxygen and trigger ROS production and lipid peroxidation.^30^ Dox-dependent iron overload is compartmentalized as iron preferentially accumulates in cardiac mitochondria. Despite this well-established close association of anthracyclines, free iron, and activation of ROS, accumulating data demonstrate that neither anti-oxidants nor some iron chelators can provide therapeutic benefits in preclinical models and clinical trials.^31–33^ Of those iron chelators tested only dexrazoxane appears to be more promising^34–36^ in both breast cancer patients and children with high risk acute lymphoblastic leukemia. Dexrazoxane has shown a significant cardioprotective effect as measured by both noninvasive testing (multiple-gated acquisition (MUGA) scan) for systolic function and improvement in clinical CHF symptoms, although the time to disease progression and the long-term event-free survival appear to be not affected.^37, 38^ This clearly suggests existence of ROS/Iron-independent mechanisms for anthracycline cardiotoxicity.

Dexrazoxane, the only Food and Drug Administration (FDA) approved effective protectant against anthracycline cardiotoxic effects, was shown to be a catalytic inhibitor of topoisomerase II (TopII).^39^ There are two TopII isozymes. TopIIα, highly expressed in cancer cells and required for cell division, is the target for anthracycline’s antitumor effect.^40–42^ However, adult cardiomyocytes express only TopIIβ, which is not required for cell division.^43^ Since dexrazoxane binds to TopIIβ and inhibits Dox-induced DNA double-strand break, it is likely that Dox causes cardiotoxic effects by targeting TopIIβ in cardiomyocytes.^39^ By binding to TopIIβ in the nucleus and stabilizing this enzyme, Dox causes continuous DNA breakdown and prevents the broken DNA double helix from repairing. Dox-induced DNA double-strand breaks and apoptosis through the p53 pathway activation. This effect could be blunted in a mouse model in which TopIIβ was genetically deleted.^39, 44^ In addition to nuclear TopIIβ, cardiomyocytes also express mitochondrial topoisomerases including TopIIβ, TopIIIα, and TopImt. TopImt is the mitochondria specific isoform and important for protecting mitochondria DNA from damage. TopImt knock-out mice are significantly more sensitive to doxorubicin cardiotoxicity and exhibit marked mitochondrial dysfunctions including impaired respiratory chain protein production and mitochondrial cristae ultrastructure organization, along with decreased O_2_ consumption and increased ROS production, eventually leading to advanced CHF and increased mortality.^45^ While doxorubicin traps nuclear TopIIβ cleavage complexes, resulting in mitochondrial DNA damage and cardiac dysfunction, TopImt is cardioprotective as it maintains normal mitochondrial DNA homeostasis and enables mitochondrial DNA to be replaced (Fig. 2).

The demonstration of the molecular targets including TopIIα, TopIIβ, and TopImt which are all related to anthracycline therapy has significant pharmaceutical and clinical implications: First, it provides the rationale for developing TopIIα-specific anticancer drugs to prevent other tissue toxicities (i.e., cardiotoxicity) in patients receiving anthracycline-based chemotherapy. Drugs that specifically target the TopIIα isozyme, but not TopIIβ or TopImt should be less cardiotoxic and, hence, more useful clinically. Second, patients with higher expression of TopIIβ in cardiomyocytes may be more susceptible to anthracycline-induced cardiotoxicity. Judicious recommendation of genetic and molecular testing could have a potential role in risk-stratifying patients when selecting anthracycline based chemotherapy regimen. Thirdly, in those high risk patients including who (1) express high levels of cardiac TopIIβ and low levels of TopImt, (2) will need higher total dose or prolonged maintenance anthracycline therapy, and (3) will need additional cardiotoxic agents, selective co-administration of the protective agent dexrazoxane could be more beneficial.

### HER2-targeted cancer therapy cardiotoxicity

Overexpression of human epidermal growth factor receptor 2 (HER2/ErbB2) in breast cancer is a poor prognostic indicator, as these tumors tend to be more aggressive and associated with higher recurrence rates.^46^ Trastuzumab (Herceptin), a humanized anti-HER2 monoclonal antibody targeting the extracellular domain of this receptor, has been shown in both the metastatic^47^ and the adjuvant^48^ setting to dramatically change the survival in HER2 positive breast cancer. The molecular mechanisms of HER2/ErbB2 signaling and related anticancer action have been well-reviewed.^49, 50^ These receptor (ErbB2/ ErbB4) are also expressed in cardiomyocytes.^51^ Activation of this pathway by growth factor neuregulin-1 (NRG-1) plays a protective role against myocardial stress.^51, 52^ The binding of anticancer drugs to HER2 receptor may disrupt this cardioprotective pathway and result in cardiotoxicity (Fig. 2). Clinical trials in the adjuvant setting reported CHF in 1.7 to 4.1% and left ventricular dysfunction in 7.1 to 18.6% of patients receiving trastuzumab, although in practice, incidence may be higher.^53^ Interestingly, emerging evidence also indicates that this pathway is critically involved in mechanisms of anthracycline cardiotoxicity. Mice with cardiac-specific overexpression of ErbB2 had a lower level of mitochondrial and whole heart ROS, as well as less myocyte death after isolation. Cultured H_9_C_2_ cardiomyocytes transfected with ErbB2 showed less cellular toxicity and produced less ROS after doxorubicin treatment.^54^ Mice with a ventricular-restricted deletion of ErbB2 (ErbB2−/−) develop chamber dilation, wall thinning and decreased contractility. Cardiomyocytes from these ErbB2−/− mice are more sensitive to Dox-induced cell death.^55^ Similarly, worsening left ventricular systolic function and survival were seen in mice when the neuregulin-1 gene was knocked out, and this is associated with the depressed activation of the ErbB2 receptor.^56^ Thus, inhibition of the pro-survival NRG-1/ErbB pathway provides a possible explanation to the finding that combinations of anthracyclines (e.g., doxorubicin) and anti-ErbB monoclonal antibodies (e.g., Herceptin) enhance antitumor efficacy but cause more pronounced cardiotoxicity than either treatment alone. However, there are noticeable differences between the anthracycline and trastuzumab cardiotoxicity: Unlike anthracyclines, there is no relationship between the accumulative dosage of trastuzumab and the probability of cardiotoxicity which is usually reversible upon cessation of drug administration and/or initiation of guideline directed medical therapies for cardiomyopathy (such as β-blockers and ACE-I). This information is clinically relevant as combination therapy using anthracyclines and monoclonal antibodies targeting ErbB2 is currently the standard of care for breast cancer patients who are HER2-positive: Adding trastuzumab to adjuvant Dox chemotherapy has significantly decreased the breast cancer recurrence risk by 50%, and mortality by 30% in HER2-positive patients. Thus, for clinical cardio-oncologists optimized treatment strategies should be developed to minimize the cardiotoxicity without significantly compromising its therapeutic benefit. Treatment duration can be adjusted and clinical responses including potential cardiotoxicity should be carefully monitored. Because the inhibition of HER2 signaling by trastuzumab in patients receiving Dox may interfere with the protective effects of NRG-1 on the anthracycline-damaged myocardium (see Fig. 2), the most-effective means to limit the cardiotoxicity is to modulate the dosages and prolong the time between Dox and trastuzumab by at least 90 days.^57, 58^

### Alkylating agents related cardiotoxicity

Alkylating agents including nitrogen mustards (i.e., cyclophosphamide and ifosfamide) and the platinum-containing molecule, cisplatin, are the oldest class of anticancer agents. They exert their action via binding to negatively charged DNA sites, causing DNA strand breaks and DNA strand cross-linking.^59^ Cyclophosphamide is a prodrug which upon activation forms an alkylating molecule that binds to DNA and causes inter-strand and intra-strand DNA breaks **(**Fig. 2). Manifestations of cyclophosphamide-induced cardiotoxicity include pericardial effusions, myocarditis, pericarditis, and heart failure which is irreversible in 25% of cases at a doses of  ≥ 1.55 g/m^2^/day. Left ventricular dysfunction develops in 7 to 28% of patients and may be dose related, occurring shortly after initial administration.^60^ Known risk factors include total bolus dose, older age, combination therapy with other cancer drugs and mediastinal radiation.^61^ In addition to vascular events such as deep vein thrombosis and pulmonary embolism, cisplatin treatment is also associated with both acute and late-onset cardiotoxicity. Acute myocardial infarction, angina pectoris, and cerebrovascular ischemia are relatively uncommon, occurring in ~2% of patients.^62^ Likely pathophysiology is multifactorial including procoagulant and direct endothelial toxic effects, as well as hypersensitivity reactions occurring during treatment. Patients could also develop subclinical abnormality in systolic dysfunction with incidence rates of 6 and 33%, respectively, 10 to 20 years after initial treatment.^63^

### Antimetabolites and antimicrotubule agents related cardiotoxicity

In cancer patients treated using 5-fluorouracil (5-FU) containing regimen, cardiac symptoms generally occur early during the drug infusion. A meta-analysis reported an incidence of symptomatic cardiotoxicity of 1.2 to 4.3% during treatment with 5-FU and suggested that the risk can be increased by continuous infusion and concurrent treatment with alkylating agent cisplatin.^64^ The most common symptom, the typical angina pectoris is reversible but myocardial infarctions have also been reported.^65^ 5-FU cardiotoxicity is relatively infrequent, independent of dosage, and may be related to a continuous infusion schedule. The presence of cardiac risk factors is not predictive.^66^ The pathogenic mechanism of cardiovascular toxicity associated with 5-FU is not completely understood; however, coronary thrombosis, arteritis, and vasospasm have been proposed as possible explanations. Additional hypothesized mechanisms are direct toxic effects of the drugs on the myocardium, interaction with the coagulation system, and autoimmune responses.^67^ 5-FU can induce apoptosis and autophagy through the production of oxidative stress in cardiomyocytes and endothelial cells.^68^ In animal model the cardiotoxicity from 5-FU and capecitabine was found to be associated with the formation of ROS, lipid peroxidation, and a rapid depletion of glutathione; the resulting increase in oxidative stress was associated with mitochondrial dysfunction (Fig. 2), which triggered caspase-3 activation and led to apoptosis or necrosis.^68^ Other studies have also shown that 5-FU can induce dose and time-dependent depletion of high energy phosphates in myocardial cells.^69–71^ In 5% of patients treated with paclitaxel, atrioventricular block, left bundle branch block, ventricular tachycardia, and ischemic cardiac events were observed, whereas asymptomatic bradycardia occurred in a variable proportion of patients (from < 0.1 to 31%).^8, 72^ Arrhythmias and conduction disorders were likely mediated by an effect of paclitaxel to accelerate spontaneous calcium release in cardiomyocytes through interacting with the neuronal calcium sensor 1, a calcium binding protein that is known to regulate the inositol-1,4,5-trisphosphate receptor.^73^ While paclitaxel itself rarely causes CHF, the combination of paclitaxel with anthracycline therapy does facilitate the anthracycline-associated cardiotoxicity, likely due to reduced anthracycline elimination resulting in higher plasma drug accumulation.^74^

### Tyrosine kinase inhibitors (TKI) and Vascular endothelial growth factor (VEGF) antibody related cardiotoxicity

VEGF is the main member of a family of seven structurally and functionally related cytokines (VEGF-A, VEGF-B, VEGF-C, VEGF-D, VEGF-E, VEGF-F, and placental growth factor. These molecules play a critical role in angiogenesis, cell survival, growth, and proliferation of endothelial cells by binding to specific receptors including VEGFR-1, VEGFR-2, VEGFR-3, and neuropilin.^75^ VEGF-A is the most representative compound and its mRNA is expressed in several tissues including the heart. VEGFR-2 is the most important receptor in mitogenesis signaling. VEGF signaling is known to play an essential role in cancer growth, invasion, and angiogenesis. Readers are also referred to the excellent reviews regarding the cellular and molecular mechanisms of TKI and anti-VEGF therapies.^76, 77^ This pathway has emerged as an important target in cancer drug development over the past decade. As such, anti-VEGF treatments including specific VEGF antibodies and VEGFR TKIs are currently the standard of care for several malignancies. Such small molecules are characterized by a targeted action^78^ on well-known proteins with important roles in cancer biology. Unfortunately, despite their “selective” action they can still cause cardiovascular complications such as arterial hypertension (HTN), QT interval prolongation, CHF, cardiomyopathy, stroke, acute myocardial infarction, thromboembolic events and cardiovascular deaths.^79, 80^ While characterization of the detailed, complex molecular mechanisms of anticancer actions of TKIs and VEGF antibodies is beyond the scope of this review (as many signaling molecules may be involved), TKI and VEGF antibody-induced cardiotoxicity can be recognized by both on-target (inhibiting VEGFR) and off-target (inhibiting other targets unrelated to VEGFR) mechanisms.

Sunitinib, an orally given small-molecule TKI commonly used to treat renal malignancies, has a long-term CHF cumulative incidence of 1.5–4.1%.^81^ The real-world experience is in fact worse, with ≈14% of patients experiencing a > 10% decline in ejection fraction.^82^ Meta-analysis from 4679 patients in 10 randomized controlled trials treated with VEGFR TKIs reported 15% deaths from myocardial infarction among all fatal adverse events (FAE) which occurred at 1.5%, and two fatal CHF cases, although hemorrhage was the most frequently (47.5%) occurring FAE.^83^ Both sunitinib and sorafenib can block multiple tyrosine kinase receptors, making it difficult to identify the on-target mechanism(s) of the well-documented side effects including CHF, hypertension, and intracranial bleeding. An off-target effect of sunitinib on ribosomal S6 kinase can increase myocyte apoptosis^84^ leading to heart failure. However, the risk of relatively specific TKIs such as axitinib on renal cell carcinoma patients was similar to that of the relatively non-specific TKIs (sunitinib, sorafenib, vandetanib, and pazopanib).^85^ Sunitinib inhibits angiogenesis by targeting the tyrosine kinase domain of VEGFR. By blocking the VEGF–VEGFR signaling pathway (on-target action) sunitinib reduces capillary density and inhibits the generation of nitric oxide (NO), thus blunting the vasodilation of NO and leading to hypertension and myocardial stress. A marked increase in systemic hypertension results in significant increases in the afterload of left ventricle and myocardial oxygen demand leading to myocardial injury/infarction and LV systolic dysfunction in vulnerable patients. The incidence of hypertension has been estimated at 15–47% with sunitinib and 17–42% in patients treated with sorafenib.^86–88^ Multiple off-target mechanisms are also recognized with sunitinib cardiotoxicity. Sunitinib can inhibit the kinase domain of platelet-derived growth factor receptor and prevent myocytes from responding to stress by secreting proangiogenic factors.^89^ Another off-target action of sunitinib, the direct inhibition of adenosine monophosphate-activated protein kinase (AMPK) which is a regulator of cardiomyocyte response to stress,^90^ is suggested to play a central role in cardiotoxicity since an adenovirus-mediated gene transfer of an activated mutant of AMPK reduces sunitinib-induced cell death. CHF may occur as a result of direct cardiomyocyte mitochondrial damage and cytochrome C-induced apoptosis.^86^ In cultured cardiomyocytes, sunitinib induces loss of mitochondrial membrane potential and energy depletion^90^ leading to cardiomyocyte dysfunction (Fig. 2). QT interval prolongation appears to be another off-target class electrophysiological effect for some of the TKIs including sunitinib.^91, 92^ If inadequately managed, these cardiovascular effects could further increase the morbidity and mortality of a high risk patient population.

Bevacizumab is a recombinant monoclonal antibody against VEGF-A which blocks angiogenesis by inhibiting the binding of the normal VEGF ligand to its receptor. This agent is approved in the United States to treat metastatic colorectal cancer, metastatic nonsquamous, non-small cell lung cancer, renal cell carcinoma, glioblastoma multiforme, and ovarian cancer. The original approval of this agent for metastatic colorectal cancer by the US FDA marked the modern era of antiangiogenic therapy for cancer patients. Bevacizumab is associated with a small increase in the risk of LV dysfunction with CHF developing in 1% by bevacizumab alone and 3% with prior chemotherapy.^93, 94^ Another meta-analysis including 3784 patients^95^ showed that bevacizumab in metastatic breast cancer increases the risk of grade 3 or 4 CHF (high grade CHF defined by National Cancer Institute common toxicity criteria NCI-CTC, version 2 or 3; http://ctep.cancer.gov) by five-fold, with an overall incidence of 1.6%. The cardiotoxicity could be due to antagonism of the VEGF-mediated angiogenesis and endothelial integrity known to protect cardiac myocytes from oxidative stress,^96^ or HTN which is a class effect of such drugs reported in every trial involving these inhibitors,^97^ as precipitation of underlying cardiac dysfunction.^96^

Understanding the molecular targeting and cellular mechanisms of cardiotoxicity of this class of anticancer agents is essential for clinical cardio-oncologists for effective patient management. Sunitinib, pazopanib, and especially vandetanib prolong the QT and therefore increase the risk of Torsades de pointes (TdP), a form of lethal arrhythmia. These drugs should only be used cautiously in the presence of a history of QT prolongation or concomitant antiarrhythmic treatments, bradycardia, or electrolyte abnormalities, while in such conditions vandetanib should be completely avoided. In the setting of TKI-containing chemotherapy it is also critical to evaluate the pro-arrhythmic effect of β-blocker therapy which is usually antiarrhythmic and especially beneficial for CHF and cardiomyopathy protection, since bradycardia from β-blocker therapy further promotes development of TdP. Consideration should also be given to the fact that while HTN develops as the most common cardiovascular side effect of the anti-VEGF treatment, and in the case of bevacizumab for metastatic colorectal cancer patients of which 20% developed grade II-III HTN (systolic BP ≥ 160 mmHg), a partial remission was observed in 75% of the hypertensive but only 32% of the normotensive patients.^97^ Those patients with grade II-III HTN also had a significantly longer progression-free survival.^98^ In the absence of guideline or data from large randomized controlled trial, individualized therapy must be formulated by cardio-oncologists to balance the effective therapeutic actions of these anticancer agents against the severity of cardiovascular toxicity profile.

### Radiation therapy related cardiotoxicity

CVD related to thoracic and mediastinal radiation therapy (RT) of cancer survivors remains the most common nonmalignant cause of morbidity and mortality.^99^ RT is associated with macrovascular, microvascular, and endothelial injury, valvular dysfunction, atherosclerosis, fibrosis, and pericardial disease including effusive or constrictive pericarditis. Left ventricular dysfunction and CHF can occur as acute radiation myocarditis but more commonly develops as a long-term consequence of fibrosis leading to ventricular dysfunction or restrictive cardiomyopathy.^100^ Overall, compared with non-irradiated patients, patients who have undergone chest radiotherapy have a 2% higher absolute risk of cardiac morbidity and death at 5 years and a 23% increased absolute risk after 20 years.^101^ In general, the tolerance dose of human myocardium is ~40 Gy.^102^ Cardiac myocytes are relatively resistant to radiation damage because of their post-mitotic state. However, cardiac endothelial cells remain sensitive to radiation, and the pathophysiology of most forms of radiation-induced cardiovascular disease (RICD) appears to be associated with damage to endothelial cells (Fig. 2). Radiation is believed to result in transient increases in oxidative stress, resulting in formation of ROSs and a subsequent inflammatory response that includes activation of nuclear factor-kappa B. Upregulation of proinflammatory pathways results in increased expression of matrix metalloproteinases, adhesion molecules, and proinflammatory cytokines and downregulation of vasculoprotective nitric oxide.^103^ Indirect evidence for radiation-induced vascular inflammation comes from numerous studies that demonstrated increased levels of the proinflammatory cytokines interleukin 6, tumor necrosis factor alpha, and interferon gamma in Japanese atomic bomb survivors.^104^

Ischemic heart disease is the most common cause of cardiac death in patients who have undergone radiation therapy. Atherosclerotic lesions in RICD are morphologically identical to those in non-irradiated vessels and are characterized by intimal proliferation, accumulation of lipid-rich macrophages, and plaque formation.^105^ Epidemiologic studies suggest a 40-year cumulative incidence rate of 24.8% for RICD, and most cases involve characteristic cardiac insults such as pericarditis, pericardial fibrosis, valvular disease, coronary disease or myocardial infarction.^106, 107^ Profound inflammation-induced by radiation injury results in the development of a diffuse, patchy interstitial fibrosis of the myocardium, as well as exudate of a variable amount of neutrophil infiltrated and protein-rich fluid within the pericardial sac. Rapid accumulation of pericardial effusion rarely can cause cardiac tamponade necessitating urgent pericardiocentesis. Chronic inflammation results in thickening of pericardium and pericardial adhesion. Clinically, diastolic heart failure or heart failure with preserved systolic function first develops when heart loses compliance from both myocardial fibrosis and pericardial thickening/adhesion. Wall motion abnormalities including both systolic and diastolic dysfunctions were found in 13, 18, and 29% of patients with a latency of two to 10, 11 to 20, and longer than 20 years after RT, respectively, vs. only 5% in age-matched controls without a history of irradiation in the Framingham study.^108^ In an autopsy series of 27 patients with RICD,^109^ 14 of the 20 (70%) available pericardium examinations demonstrated significant pericardial diseases (effusions, constrictions or both). In another necropsy series of 16 patients with known RICD^110^ pericardial thickening was found in all cases (100%).

Implementation of dose reducing techniques for RT has significantly reduced the acute pericarditis which usually occurs days to weeks after therapy at doses higher than 40 Gy. The dose-dependent (usually ≥ 50 Gy), chronic pericarditis is the most common cardiac complication from RT, which usually occurs from 3 months to over a decade with 1 year being the median time. The radiation-sparing techniques routinely used nowadays have dramatically reduced the incidence of symptomatic, chronic, delayed pericarditis from ~20% in the 1970’s to about 2.5%.^111^ Dose-volume histogram calculation for the heart, prone position, and deep inspiration breath hold can all lower the risk of direct damage to the heart. Radiation is known to induce oxidative stress with generation of ROS which is thought to play a key role in the transition from acute inflammation to chronic inflammation and fibrosis.^112^ These radiation-sparing measures may also prevent the additive or synergistic effect of ROS with other cancer therapy agents such as anthracyclines and antimetabolites as discussed above.

## Multi-disciplinary approaches toward individualized cardio-oncology care

Guideline directed principles^113–115^ of diagnosis, prevention, and treatment should be followed with particular attentions paying toward individual patient clinical profiles including age, gender, pre-existing cardiovascular risk factors, type of cancers and treatment regimen. Clear communication among a large multidisciplinary team (Fig. 1) including cardiologists, oncologists, imaging specialists, clinical pharmacologists, the patient, and their family is essential for many life-modifying decisions, and this often requires periodic reconsideration during a course of therapy. Adding to the complexity is the fact that many decisions must be based on limited evidence, and in the context of rapidly evolving cancer therapeutics, experience and expert opinion become increasingly important. Cardio-oncology care should include primary prevention of cardiovascular complications in “high risk” patients with aggressive risk factor modifications and ongoing monitoring early toxicities, effective treatment of complications that have already developed and active prevention of worsening complications, pre-operative assessment of cardiovascular risks for cancer surgeries, and investigation of possible cardiac invasion from malignancy. A multi-disciplinary approach to the management of cardiomyopathy and CHF in cancer therapy patients will be elaborated below.

### Cardiomyopathy and CHF

**Fig. 3:**
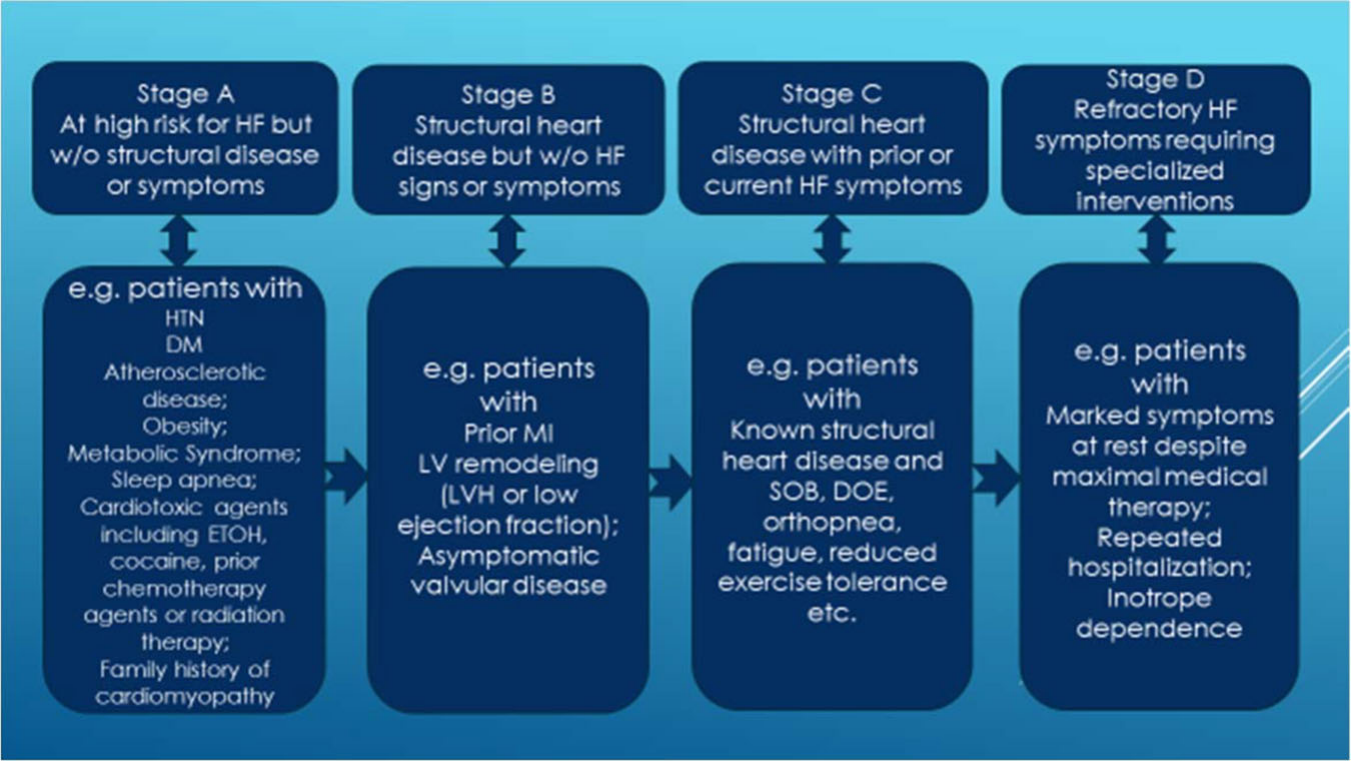
Stages in the development of heart failure. Abbreviations: HF, heart failure; w/o, without; HTN, hypertension; DM, diabetes; MI, myocardial infarction; LVH, left ventricular hypertrophy; SOB, shortness of breath; DOE, dyspnea on exertion. Modified from ref.^114, 115^

Cardiomyopathy implies structural heart abnormalities. CHF is a clinical syndrome characterized by typical signs and symptoms.^113^ According to American College of Cardiology/American Heart Association/Heart Failure/Society of America guideline staging,^114, 115^ symptomatic ventricular dysfunction is often an avoidable late stage in a chronic process including cancer-related treatment (Fig. 3). Overwhelming evidence supports the notion that CHF can be prevented and the onset delayed by modifying risk factors (stage A) for CHF and treating asymptomatic left ventricular systolic dysfunction (stage B). Because the diagnostic suspicion relies on clinical symptoms and signs, medical history and physical examination continue to be vital. CHF can be further classified^113^ into heart failure with preserved EF (HFpEF), heart failure with medium range EF (HFmrEF), and heart failure with reduced EF (HFrEF), according to the left ventricular ejection fraction (LVEF).

### Diagnostic modalities

The first step in preventing heart failure should be a careful clinical assessment of modifiable and non-modifiable risk factors, during a comprehensive clinical assessment. During any baseline assessment, it is also important to clarify the cancer therapy that is planned by the oncology team. Diagnostic workup includes biomarkers and imaging modalities. Myocardial injury from cancer treatment-induced cardiotoxicity releases cardiac troponins which can be detected long before any reduction in LVEF has occurred. In a multicenter study^116^ of breast cancer patients undergoing doxorubicin and trastuzumab therapy, the associations of 8 biomarkers including the ultrasensitive troponin I (TnI), high-sensitivity C-reactive protein (CRP), N-terminal pro–B-type natriuretic peptide (NT-proBNP), growth differentiation factor-15, myeloperoxidase (MPO), placental growth factor (PlGF), soluble fms-like tyrosine kinase receptor (sFlt)-1, and galectin (gal)-3 with the successive development of cardiotoxicity were analyzed. The most important risk of cardiotoxicity was associated with TnI change in absolute values. The risk of cardiotoxicity was 46.5% in patients with the largest changes in both TnI and MPO. Increased brain-type natriuretic peptide (BNP) levels can detect chemotherapy-induced LV dysfunction in both adult and pediatric populations.^117, 118^ Although significant controversies exist regarding the predictive value of BNP since many studies failed to find a correlation between the level of BNP and the degree of reduced LV systolic function (HFrEF), this biomarker (BNP or NT-ProBNP) is required in the new ESC guideline for the diagnosis of HFpEF and HFmrEF.^113^ Regarding the diagnostic imaging tests, transthoracic echocardiography (TTE) is the method of choice for determining systolic and diastolic function in patients with HF. Two dimensional (2D) and especially Doppler strain imaging can reliably detect myocardial deformation variations which may develop early during anticancer therapy. The speckle tracking imaging can most accurately measure the reduction in peak systolic global longitudinal strain which usually precedes the development of systolic dysfunction and syndromic CHF. Three dimensional (3D) echocardiography can detect subtle changes in LV dysfunction that could be missed by 2D study. Cardiac magnetic resonance imaging (MRI) is considered the gold standard for the evaluation of the volume, mass, and EF of both ventricles, and the preferred method for imaging the right ventricle and for patients with complex congenital heart disease. It is the best imaging method to detect sub-endocardial damage and myocardial fibrosis. In particular cases such as patients with older models of implantable devices (defibrillators, pacemakers etc.) when MRI is contraindicated, single-photon emission computed tomography, positron emission tomography, and noninvasive coronary angiography with multidetector computed tomography can be useful. Historically, MUGA radionuclide imaging formed the mainstay of cardiac monitoring in patients receiving potentially cardiotoxic therapy. While it can reproducibly measure LVEF than 2D-TTE,^119^ and reductions during chemotherapy have been associated with adverse outcome, it may result in significant cumulative radiation exposure (~10 mSv per study) yet provides only limited information on cardiac structure and diastolic function, thus limiting repeated imaging.

### Treatment of heart failure from stage A to D

In Stage A, all risk factors should be aggressively treated. Many trials show that control of hypertension will delay the onset of CHF and some also show that it will prolong life.^120–122^ This is especially important in cancer patients treated or to be treated with anti-VEGF regimen which is known to cause significant treatment-related HTN. Other conditions that may lead to or contribute to CHF, such as obesity, sleep apnea, diabetes mellitus, hyperlipidemia, and tobacco use should be controlled. The cardiotoxicity of known chemotherapy agents should be carefully evaluated at this stage. In cancer patients with known family history of familial cardiomyopathy (usually at relatively young age) genetic testing might be considered. In those with known disease gene carriers less cardiotoxic chemotherapy regimen with closer monitoring of cardiotoxicity should be adopted. If structural damage has already occurred (stage B), guideline recommended β-blocker, ACE-I or Angiotensin Receptor Blocker (ARB) should be administered in the absence of contraindication. Treatment of symptomatic patients with significant structural heart damage (stage C) should include all options for stages A and B plus aldosterone receptor antagonists, as well as the combination of hydralazine and isosorbide dinitrate, and the judicious use of diuretics and digoxin. Patients in stage C disease may benefit more from a combination drug Entresto (a neprilysin inhibitor, sacubitril, and an ARB, valsartan) than the traditional ACE-I or ARB. Critical decision will need to be made about continuation or withholding of chemotherapy after carefully balancing the risks of avoiding cancer treatment against further cardiac toxicity. Sudden death risk in patients with stages B and C disease on optimal medical treatment should be discussed, and in those patients with favorable long term survival ( ≥ 1year) implantable cardioverter defibrillator can be recommended. Resynchronization therapy should also be considered in the above stage C patients with a reasonable survival ≥ 1 year. If patients are progressing to stage D disease (may also be called “refractory”, “advanced”, or “end-stage”) treatment options are primarily of fluid restriction, inotropic agents, mechanical circulatory support, heart transplantation (HT), and palliative or “end-of-life” care. Regarding heart transplant, the outcomes of 232 chemotherapy related cardiomyopathy patients had similar 1-year, 2-year, and 5-year survivals (86 vs. 87%, 79 vs. 81%, and 71 vs. 74%; *P* = .19) comparing with other non-ischemic cardiomyopathy patients,^123^ despite a higher rate for post-transplant infection (22 vs. 14%, *p* = 0.04) and malignancy (5 vs. 2%, *p* = 0.006) in the chemotherapy-induced cardiomyopathy group. In the most recent ISHLT position paper no specific cancer-free interval is recommended before listing for HT, as long as the chance of tumor recurrence is deemed low with negative metastatic work-up.^124^ Due to the extremely poor survival^125, 126^ with 1 year mortality of ~90% or higher, aggressive chemotherapy for cancer patients with stage D heart failure on inotropic agents likely offers no benefit. Discussions of individualized care plans with active involvements of family members and social worker supports usually result in high patient’s satisfaction. At any stage, the key to successful management is the close collaboration between cardiologists and other experts, including primary oncologists, general practitioners, pharmacists, dieticians, physiotherapists, psychologists, palliative care providers, and social workers^113–115^ (Fig. 1).

## Conclusions

This review has highlighted the increasing importance of CVD management in cancer patients, with particular attention toward understanding the molecular and cellular mechanisms of cardiovascular toxicity from cancer therapy, and emphasized the important role of multi-disciplinary cardio-oncology service team in this complex and evolving process. While contemporary cancer treatment strategies have resulted in dramatic improvement of patients surviving a diagnosis of cancer for many years, such gain in quantity, as well as quality of life may be offset by the mortality and morbidity from therapy-related side effects on the cardiovascular health. Unfortunately the spectrum of cardiovascular complications associated with the ever-changing cancer therapy is not expected to decrease in the foreseeable future. Burden of heart failure after cancer treatment remains excessively high, and even the most advanced level of cardiac care offered to these patients remains suboptimal. This is at least in part due to the challenge from a number of factors somewhat unique to the field, including the complex and growing array of malignancies, novel anti-cancer agents, new cardiac imaging modalities, absence of targeted cardioprotective treatments, and lack of coordinated care of patients with cancer and CVDs. How can we accurately predict an individual patient’s cardiotoxicity with “standard of care” chemotherapy regimen remains to be the most challenging question, among others such as the optimal strategy for cardiotoxicity monitoring and management. As such, future evidence-based research to improve the care of patients of cancer survivors should focus on the following areas: (1) improvement in our understanding of the precise molecular and pathophysiological mechanisms of cardiotoxicity; (2) improvement in risk prediction that allows targeted treatment and avoidance of unnecessarily burdensome therapies for patients most likely to develop cardiotoxicity; (3) randomized controlled trials comparing surveillance frequency for cardiotoxicity prevention and superiority of different treatment strategies on minimizing and preventing cardiovascular complications. The widely advocated precision medicine encompassing “big data” and omics including pharmacogenetics-pharmacogenomics^127, 128^ would fit perfectly well to the urgently needed area of cardio-oncology research and clinical care. Finally, the multidisciplinary approach of cardio-oncology service with close collaborations among oncologists, cardiologists, and other allied health care professionals will be essential in the development and promotion of clinical care models to improve long-term outcomes of cancer treatments and cancer survivors.
